# Facilitators of person and relationship‐centred care in nursing

**DOI:** 10.1002/nop2.1083

**Published:** 2021-09-30

**Authors:** Tony Ryan

**Affiliations:** ^1^ Division of Nursing & Midwifery, Health Sciences Scholl Faculty of Medicine Dentistry & Health University of Sheffield Sheffield UK

**Keywords:** nursing, person‐centred care, relationship‐centred care

## Abstract

**Aims:**

To provide an expert overview on the current state of evidence as it relates to person and relationship‐centred care.

**Design:**

Review and commentary.

**Methods:**

The paper was prepared in order to contribute to a Consensus Development Project. It is based upon a scoping review with additional theoretical material used to supplement the narrative. The content is limited to that person and relationship‐centred literature as it relates to nursing practice and policy.

**Results:**

There is compelling evidence in favour of nurses pursuing person and relationship‐centred policies and practices. Organizational and individual factors contribute to the successful implementation of person and relationship‐centred care. These include conditions that enable nurses to provide high‐quality care (resources, clinical supervision and security) and include training and development, a biographical approach to care and those care environments centred on innovation and person‐centred care processes.

## 1 BACKGROUND AND INTRODUCTION

Person and relationship‐centred care provides the bedrock of professional practice, policy and education in a nursing context. The World Health Organisation (WHO) has cited person‐centred care (or people‐centred) as a major global objective, enabling individuals and communities to take control of their own health (WHO, 2015). The International Council of Nurses retains the achievement of person‐centred care as one of its 10 strategic priorities. Others have noted the significance that person‐centred care plays in the policy landscape of countries all around Europe, citing access to services, continuity of care, involvement in decision making, effective treatment and dignity and respect as the cornerstone of healthcare delivery (Paparella, 2016). In the UK, professional practice standards are most readily articulated by the Nursing and Midwifery Council (NMC, 2018), where person‐centred care is a pivotal theme. A number of NICE practice guidelines cite the importance of person‐centred care in involving people in healthcare decisions (NICE, 2015, 2016, 2018).

Person‐centred care as a “way of working” within healthcare has become well established in recent years. Notable contributions to the field have come from Kitson et al. (2013), Sharma et al. (2015) and Waters and Buchanan (2017). Despite a lack of Consensus on what is meant by the term person‐centred care, there remain a number of common threads or conceptually consistent patterns within the literature and these will be further elaborated upon later in this paper. For now, however, the term person‐centred care might usefully be articulated as moving care beyond the individual's disease to ensure that care work focuses “on the needs of individual. Ensuring that people's preferences, needs and values guide clinical decisions, and providing care that is respectful of and responsive to them… Health and wellbeing outcomes need to be co‐produced by individuals and members of the workforce working in partnership” (HEE, 2021). Two major models exist within the nursing field, these are: The Person‐Centred Nursing Framework (McCormack & McCance, 2011) and the Senses Framework (synonymous with relationship‐centred care) (Nolan et al., 2004). Person‐Centred Nursing Framework (PCNF) recognizes the centrality of person‐centred nurse competences, person‐centred processes and the care environment in achieving satisfaction and involvement in care, well‐being and a therapeutic culture. Both the PCNF and RCC focus on the notion that the relationship between person (or patient) and nurse is central to an enriched care experience. Both also recognize the role played by wider family and other support, in the form of people or organizations. Relationship‐centred care, however, places emphasis upon ways in which nurses themselves experience the care encounter and environment, recognizing explicitly that the healthcare workforce is unlikely to provide “the highest quality of care, unless they have a positive predisposition towards such care” (Nolan et al., 2004, pp. 48). Consequently, the set of conditions (clinical supervision, job security and resources), which come to “frame” nursing work and those relationships between nurses and the recipients of care (care processes), become significant.

The paper was prepared in order to contribute to a “public conversation” about a range of important nursing issues, as part of a Consensus Development Project. The paper is based upon a review of existing literature, gathered as part of a scoping exercise. Additional theoretical material was used to supplement the narrative. The material included in the paper is limited to that person and relationship‐centred literature as it relates to nursing practice and policy. It recognized that some crossover inevitably exists. The paper will address key themes, evidence supporting person and relationship‐centred care as a policy and practice intervention, before going on to highlight those individual and organizational facilitators important in its implementation.

## 2 KEY THEMES

The academic field is broad, and whilst there remains a great deal of crossover, there exists some contention about how we might conceptualize and understand the nature of person and nurse interaction. How this might be operationalized, evaluated and promoted is also the subject of some debate. That aside, this review revealed five key indicators of person and relationship‐centred care as practiced by nurses. These are:

1. *Building Therapeutic Relationships:* ensuring that the nurse and the person/patient are able to build a relationship that enables close and productive working on an equal level (Sharma et al., 2015). Therapeutic relationships provide mutual benefit and enable the nurse to understand the social and psychological consequences of their condition (Larkin et al., 2019), and that continuity is an important element of care (Nolan et al., 2004).
2. *Maintaining Identity:* the role of the nurse in understanding the person in the context of their life and helping to provide care that seeks to maintain this. Such practice recognizes the uniqueness of each person (Jakimowicz & Perry, 2015). Nurses and other healthcare professionals should ensure their practice is based on methods of gaining access to knowledge and experiences of the person that they care for (Sharma et al., 2015).
3. *Sharing Power*: nursing seeks to provide care that is consistent with the values and wishes of the person, through the facilitation of decisions with the person, rather than for the person. Such practice is individualized and meaningful to care recipients (Waters & Buchanan, 2017).
4. *Engaging with people in a holistic manner*: Recognizing that the person exists beyond the condition or disease. Understanding that care is not limited to physical aspects of care, although these are important. Seeking to address the social, psychological, spiritual, sexual and emotional consequences of the condition (McCance et al., 2011).
5. *Relationships as significant:* Understanding that the person does not exist in isolation, but that they are part of a wider network of family, friends and community. Also, recognizing that the nurse and other HCPs form part of the network and whose needs are also important (Nolan et al., 2004).

## 3 WHAT EVIDENCE IS THERE TO SUPPORT PCC/RCC INTERVENTIONS?

There exists a wide range of methodological approaches to the evaluation of person‐centred/relationship‐centred care interventions and the assessment of impact on people who use services and the nursing workforce. These range from experimental and quasi‐experimental methods to appreciative inquiry. Interventions also vary in type and nature. Evaluation of person‐centred and relationship‐centred care interventions focus on both the measurement of person/patient outcomes, as well as those for the nurse, wider team and family. Evaluations of specific interventions are often framed within a disease or condition specific setting (for example, dementia). It should be noted that in the literature there is an emphasis on interventions in acute or residential/nursing home settings. This section is divided into three. First, those papers evaluating the implementation of person and relationship‐centred nursing models will be addressed. Second, literature focusing on “getting to know the person” will be evaluated, before turning attention to those papers evaluating person and relationship‐centred models as a complex/multi‐component intervention.

### 3.1 Evaluation of person‐ and relationship‐centred models

Interventions are described as complex in nature, comprising a range of changed practice and organizational‐based activities, with a focus on cultures of care. Most prominent in the evaluation evidence is the work associated with person‐centred nursing framework (PCNF) and relationship‐centred care, principally McCormack and McCance (2011), Nolan et al. (2004) and Smith et al. (2010). The practice development model forms part of this and has demonstrated favourable outcomes for people using healthcare services as well as families and nurses themselves. Practice development is operationalized through “sustained and continuous quality improvement” (McCormack & McCance 2006) and sees change as ongoing rather than being achieved through one‐off events. McCormack and McCance (2011) utilized a programme aimed at promoting person‐centred nursing practice in this way, and a number of publications are associated with this work. The core elements feature: promoting person‐centred understanding, developing a shared vision, determining the quality of the user experience, systematically developing practice and celebrating success (McCance et al., 2013) and transforming practice (McCormack et al., 2010). Furthermore, change, as represented through this body of work, is achieved through the provision of a series of workshops, ongoing negotiation, collaboration, individual facilitation and reflection. The work has taken place in acute and residential settings. The outcomes demonstrate considerable impact upon nursing workforce approaches and subsequent positive effects on the quality of care. In particular, a practice development methodology allows nurses to engage and build relationships across teams, whilst supporting nurses to identify aspects of the care environment that inhibit the development of person‐centred practice.

Smith et al. (2010) utilize a participatory action research and appreciative enquiry in the establishment of relationship‐centred care for older people in acute settings. This work too is located in a wider programme of activities to promote leadership, specifically aimed at promoting compassionate care. Outcomes include improved communication between service users, families and nurses, improved access to information for families and better understanding for staff of what works in clinical practice. Dewar and Mackay (2010) also note factors that might inhibit the development of person‐ and relationship‐centre care.

Further evidence of this “comprehensive” approach to development of person and relationship‐centred care can be seen in the work undertaken by Mike Nolan and colleagues. Devised a means to both recognize the centrality of interdependence and to establish social conditions consistent with providing opportunity for genuine caregiving relationships through the recognition of a sense of: security, belonging, continuity, purpose, achievement and significance (Nolan et al., 2004). The Senses Framework has been identified as a means for understanding service user, family and nurse satisfaction with community‐based dementia services, highlighting closer working relationships, continuity of care and carer satisfaction (Ryan et al., 2008). The Senses Framework has also been used to demonstrate the value of a person or relationship‐centred approach at the end of life for people with dementia (Watson, 2019) and demonstrated an increased likelihood that care is aligned with the needs of older people in care homes (Brown Wilson et al., 2013).

### 3.2 Getting to know the person

Interventions are often located within key transitions in the person's encounter with a care setting or intervention (e.g. admission). Typically, person/relationship‐centred care interventions are underpinned by activities and practices that seek to understand the person or patient. In particular, these “biographical” approaches provide the platform from which care can be organized. This approach is identified in a number of studies, including for example patients experiencing heart failure (Ekman et al., 2012), older people (McCance et al., 2013), diabetes care (Zoffmann et al., 2008) and people with dementia (McKeown et al., 2010). This pivotal feature of person/relationship‐centred approaches to care is also a feature of some of the models identified within the nursing field, such as McCormack & McCance's notion of *working with the patients beliefs and values* (McCormack & McCance, 2011) and the idea of *continuity* as part the Senses Framework (Nolan et al., 2004). For example, the Senses Framework has been used to inform biographical practice in residential care (Brown Wilson et al., 2013), whilst a person‐centred nursing approach has also been employed to build a narrative approach (Buckley et al., 2018).

### 3.3 Person and relationship‐centred care as a complex/multicomponent intervention

A number of papers have demonstrated the positive outcomes of multi‐component interventions in a range of settings, often evaluated via experimental, quasi‐experimental or mixed method study designs. Such studies provide descriptions of interventions seeking to transform assessment and care management practices. Such practices may be more focused on the person's psychosocial or spiritual needs at the end of life (Brännström & Boman, 2014), their fears after surgery (Olsson et al., 2016), social and relational issues pertinent to a proposed treatment plan, for instance during a cancer diagnosis (Hansson et al., 2017). Other studies include a combination of narrative‐based approaches with goal planning (Hansson et al., 2016). Alongside enhanced assessment practices, studies also describe improved forms of communication and patient involvement, for example the closer and increased frequency of nurse communication within the Brännström & Boman study who utilized a Six S model to frame conversations (self‐image, self‐determination, social relationships, symptom control, synthesis and surrender).

Studies of this nature have also sought to combine staff training with another aspect of care. For example, the PerCEN trial evaluated person‐centred care training, alongside improvements in the shared and public spaces in residential and nursing home care environments for people with dementia (Chenoweth et al., 2014, 2015). Similarly, other multi‐component interventions have been evaluated, combining training with other aspects of the care environment (Griffiths et al., 2019). A range of outcomes has been identified, ranging from reduced length of stay (Ekman et al., 2012), improved health related quality of life and reduced burden (Brännström & Boman, 2014; Hansson et al., 2017), reduced agitation for those people with dementia who were the recipients (Ballard et al., 2018) and reduced use of restraint (Jacobsen et al., 2017).

Despite a wide‐ranging literature, there remain some who argue that the rigorous evaluation of person and relationship‐centred interventions is somewhat mixed. Olsson et al. (2013) have argued that more research is needed to ensure what they see as a more robust evidence base. Sharma et al. (2015) also note that the evidence base requires development, but that there exists a compelling case for nurses to build therapeutic relationships to ensure partnership working and improve well‐being. Notwithstanding these observations, it remains the case that there is convincing evidence from non‐experimental sources that person and relationship‐centred care provide the basis for nursing care in a contemporary healthcare system. All three nursing models (PCNF, Senses Framework and Compassionate Care) have evolved alongside considerable experience and evidence drawn from non‐experimental sources, such as qualitative research papers, to demonstrate feasibility, acceptability and improved outcomes.

## 4 FACILITATORS OF PERSON AND RELATIONSHIP‐CENTRED CARE

### 4.1 Nurse characteristics

Much is presented in the academic and practice‐based literature about individual nurse preconditions that mean that person‐centre practices are most likely to be demonstrated. McCormack and McCance (2011) describe these as nurse attributes and provide a fundamental building block to the PCNF model. Such attributes will be discussed in relation to other evidence below, but there is overwhelming evidence to suggest that such individual level “characteristics” are significant.

#### 4.1.1 Professional competence

Competence is a central feature in much of the literature, none more so than within McCormack and McCance's PCNF (2011). Although contested at times (McCormack et al., 2010), there is little debate about the significance of professional competence in being able to practice person‐centred nursing care. The PCNF model notes at the very least, competence should be demonstrated through the meeting of regulatory requirements. More than this, however, the PCNF model describes the advanced skills associated with both technical and “non‐technical” aspects of competence as being central. Others have observed professional competence in specific settings and noted the range of competences present when establishing person‐centred nursing care. Jakimowicz and Perry (2015) point to the range of competences, such as high level clinical reasoning, decision making, ethical awareness, altruism as well as the capacity to perform highly skilled technical aspects of practice as important individual characteristics in the development of a therapeutic relationship through person‐centred care (Jakimowicz & Perry, 2015).

#### 4.1.2 Communication and interpersonal skills

Communication and interpersonal skills are prominent in the literature as a particular competence, and this is of concern in those fields of nursing care that prompt the need to share information that is both complex and sensitive. Larkin et al. (2019) cite communication practices with people at the end of their life as a critical field of person‐centred practice. Managing sensitive conversations with families in end of life care settings is viewed as highly skilled, especially when balancing notions of truth and hope (Larkin et al., 2019). McCormack and McCance (2011) also view effective communication as being a central pre‐requisite and the ability to communicate at all levels a desired competence. Similarly, a need to be competent in undertaking close communication practices with people in care and their families is implicit in the compassionate care model (Smith et al., 2010). Others note interpersonal communication within healthcare teams themselves as important in maintaining an environment where information is shared to assist in the provision of person and relationship‐centred care (Ryan et al., 2008).

#### 4.1.3 Commitment

Maintaining a conscious effort to sustain person and relationship‐centred practice has been established as an individual condition. When such effort to maintain this approach is missing, the likelihood that person‐centred care will be displayed is diminished (Moore et al., 2017). Rooted heavily in the wider literature about the nature of nursing and caregiving, McCormack and McCance (2011) describe intentionality, to do what is right for the patient, as a fundamental pre‐requisite, whilst others note a lack of motivation to achieve person and relationship‐centred care as a significant barrier (Kiwanuka et al., 2019).

#### 4.1.4 Resilience, awareness and reflection

There is evidence that nurses working in a person and relationship‐centred way require a set of personal skills that are aimed at protecting themselves, promoting responsiveness to the conditions within which they are working as well as enhancing self‐development. The compassionate care model identifies a number of these characteristics as part of the personal attributes of nurses (Dewar & Mackay, 2010). Self‐awareness is identified by McCormack and McCance as a feature of the person‐centred nursing model. The value of self‐awareness in the context of person‐centred care is emphasized when we consider that it contributes to: the capacity to develop therapeutic relationships, enhanced understanding of self and others, communication skills and the skilled management of difficult care situations (Rasheed et al., 2019). Zoffmann et al. (2008) note the significance of reflection as a means of rarefying the person/service user's voice in diabetes care.

Whilst such individual characteristics are important and it is recognized that these play a pivotal role in the nurses capability to practice in a person and relationship‐centred way, it is also important to recognize that they exist within a wider set of organizational, historical and resource driven contexts. With this in mind, the focus for much of the remainder of this paper are those organizational conditions that facilitate or inhibit person and relationship‐centred care practices.

### 4.2 Organizational level characteristics

The evidence relating to the role that organizations play in the establishment and maintenance of person and relationship‐centred care is complex and broad. Below is a summary of the primary organizational conditions identified as being important. It is significant that these organizational conditions can provide the “platform” for individual practices and the development of nurse capabilities and competences.

#### 4.2.1 Resources: Staff and physical space

A cornerstone in the provision of person and relationship‐centred practice; resources include a number of aspects of the organization identified as being important facilitators. Nurse time is often cited as a key part of this, and viewed as essential in enabling the development of relationships, attending to the complexity of care and developing new skills (Larkin et al., 2019; Moore et al., 2017; Ryan et al., 2017). McCormack and McCance point to the provision of appropriate skill mix, arguing that the balance between registered and unregistered nurses in a given environment need to be both sufficient and appropriate to meet with the challenges of complexity and acuity of the patients (McCormack & McCance, 2011). The physical space available to people who are cared for, and the nurses caring for them, has also been shown to be an important facilitator of person and relationship‐centred care. In a large cross‐sectional study, Sjögren et al. (2017) noted that adequate and pleasing physical space enabled shared and participatory care and communal activities in residential settings (Sjögren et al., 2017). Others note the significance of positive physical spaces as an organizational facilitator (Hunter et al., 2016).

#### 4.2.2 Leadership

The presence of leaders at a local and strategic level who regard person and relationship‐centred care as essential, and who seek to operationalize this, is viewed as a central feature in its implementation and maintenance. Role modelling has been identified as a specific example. In studies looking at person‐centred practice in ICU, having a leader who is able themselves to practice in a person‐centred way is viewed as a key mechanism (Kiwanuka et al., 2019), and an important barrier where this was not the case. Leaders who espouse person‐centred practice and who are able to energize and motivate teams in this way have also been identified as significant (Sharma et al., 2015). Evidence suggests that nurse “middle managers” play an important role in setting the operational tone for a care environment and knowing the difference between “hands on” and “heads on” work (Lalleman et al., 2017). Importantly, leaders are also viewed as those who are able to “give permission” for team members to focus on those aspects of person and relationship‐centred tasks and are also viewed as key facilitators (Waters & Buchanan, 2017).

#### 4.2.3 Person‐centred organizations

Much is written on this matter, and it is sometimes unclear as to what this might mean in practice. There are a number of material conditions seen to facilitate person and relationship‐centred care, and these relate to systems and procedures. Sharma et al. (2015), for example, suggests that the presence of mission statements alone does not necessarily translate into meaningful change for people. A sense of security is one of the Senses Framework and in essence means providing employees (nurses) with some of the essential components of being a practicing professional: job security, be free from threat or rebuke, to have the emotional demands of work recognized (Nolan et al., 2004). Further, continuity of care has already been noted as an important cornerstone of good practice in this regard, and many have noted that without the infrastructure to achieve this good relationships between people and nurses will not endure (Larkin et al., 2019; Waters & Buchanan, 2017). Additional observations are made about technical support, such as IT systems capable of capturing relevant person‐centred information. Such systems are viewed as being essential in helping staff to achieve continuity (Sharma et al., 2015).

#### 4.2.4 Person and relationship‐centred culture

The two primary models in the field, Person Centred Nursing and the Senses Framework, have been discussed at length above. These models are notable in their main aim of providing evidence and the need to establish a coherent and comprehensive approach to the ways in which routines; values, structures and attitudes result in good practices and care experiences. Nolan et al. (2004) speak of “enriched” environments; McCormack and McCance refer to these as “person‐centred cultures.” The road to achieving such environments is complex and in some cases requires long‐term sustained change. Two examples will help. McCormack and McCance refer to the use of a practice development framework to enable cultural change. The level of detail required to explore this is beyond the scope of this report, but essentially skilled facilitation in areas such as challenging contradictory practices, reflection, developing collective awareness, widening participation and working with values and beliefs. Their work (along with that of others) contains evaluation material demonstrating sustained change using these approaches (McCance et al., 2013; McCormack et al., 2010, 2011). Nolan et al, through a series of papers and evaluation activities, highlight the Senses Framework (as discussed), but other aligned projects note how cultures of care influence the likelihood of achieving improved outcomes for people using and working in care services. Ryan et al. (2008) note how “enriched” environments can be achieved in dementia services through open communication, collective reflection, autonomy, innovation and continuity of relationships. Patterson et al. (2011) carried out a large‐scale study of acute care for older people and identified a preponderance of the value of measuring tasks and clinically determined outcomes, top‐down transactional leadership and short‐term “quick fixes” as being associated with “impoverished care environments.” On the other hand, those “enriched environments” are characterized by person‐centred outcome measures, shared leadership and collective decision making, a focus on long‐term outcomes and experiences being used to measure success (Patterson et al., 2011). These aspects of “enriched environments” are not dissimilar to what McCormack and McCance might call “person‐centred culture”: shared power and decision making, innovation, an absence of horizontal and hierarchical bullying and a learning culture (McCormack & McCance, 2011).

## 5 DISCUSSION

The evidence provided here helps in establishing the legitimacy and central importance of person and relationship‐centred care in nursing practice and policy. The central role with wider policy and practice recommendations of this approach to nursing also adds further credence. The evidence above also provides some insight as to why the conditions for establishment and maintenance of person and relationship‐centred care as a form of practice are not always present. As such a number of organizational and practice‐based recommendations can be proposed. From an organizational perspective, environments that seek to value the people who work within them are more likely to be able to deliver person‐ and relationship‐centred practices (Nolan et al., 2004). Security of tenure, reliable career trajectories, effective and comprehensive clinical supervision, established continuing professional development (CPD), open communication with leadership structures and being part of a network of peers, are some of the ways in which this can be established. Similarly, staffing and resourcing issues sit at the very heart of the ability to practice in a person and relationship‐centred way (Ryan et al 2017). Without time, nurses cannot dedicate themselves to understanding the emotional, social, psychological and biographical aspects of the person for whom they care. Without time, nurses are also themselves burdened by non‐patient‐centred activities. Indeed Patterson et al. (2011) highlight a range of organizational aspects that hinder or facilitate the potential for relational care to flourish, including the overuse of metrics to measure success. Environments that do not innovate are also associated with practices that continue to service institutional needs (McCormack & McCance, 2011). Much emphasis in the evidence is placed upon those organizations and environments that welcome and value change as being more person and relationship centred. Through such means, teams of people (nurses) are able to work to develop in line with the needs, wishes and aspirations of the people who use services.

Practice‐based recommendations have also emerged. Evidence would suggest that the subjective, relational and personal narrative of people who use care is important in establishing person and relationship‐centred care (Ekman et al., 2012; McKeown et al., 2010). Healthcare organizations should seek to establish and improve mechanisms for collecting “person‐centred” information and promoting continuity of care. Assessment practices should focus on the collection of information that has a “biographical” focus. This would enable nurses to understand the whole person and establish goals that are consistent with the experience and values of that person (McCormack & McCance, 2011). Care should be focused around long‐term engagement between nurses and the people that they care for, rather than the episodic basis upon which much is currently arranged. Both of these changes would also help to facilitate the therapeutic relationship.

Much is made in this paper of the importance of individual competences. McCormack and McCance (2011) and the PCNF stress these attributes. Implicit in this appraisal is the need, therefore, to prepare a workforce fit for person‐centred practice. The need for a nursing curriculum and comprehensive continuing professional development programme to support the skills and competences required for person and relationship‐centred care is clear. Training‐based interventions can help to provide nurses with the individual skills to practice in a person and relationship‐centred way (Chenoweth et al., 2015). Others note that individual competences, facilitated through CPD, are interdependent on skills, leadership and workplace culture (King et al., 2020). Education and training for pre‐registration nurses, aimed at establishing awareness of such practices are consistent with NMC requirements and should be given priority within the University curricula.

The review undertaken as the basis for this work identified a great deal of high‐quality research being carried out in the field, and this continues to evolve. However, much of the evidence (although not all) relates to acute and secondary care environments. The growing emphasis upon the community and home environment as a place where care is experienced has highlighted an urgent need to develop the field within this space. As such, this paper has highlighted the need to explore these issues within community and domiciliary settings. Some have noted (Olsson et al., 2013) that the evidence base in the field is weakened by poorly designed studies, especially those of an experimental nature. Notwithstanding this observation, the work here indicates that the complex nature of care experiences and the specific mechanisms that are present when person‐ and relationship‐centred care is being experienced remain to some extent unknown. This would suggest that there is an absolute clear need to continue to explore the subject from an experiential standpoint, subsequently maintaining a robust qualitative component of the research agenda.

## 6 CONCLUSION

A range of evidence is presented here, drawn from diverse methodological positions. Those factors that can act as facilitators of person‐ and relationship‐centred care extend beyond the skills and competences of individual nurses to include a range of organizational and workplace factors. There is evidence to assist in guiding organizational and educational policies and practices that can promote the implementation of person‐ and relationship‐centred care.

## CONFLICT OF INTEREST

None to declare.

## AUTHOR CONTRIBUTIONS

All authors have agreed on the final version and meet at least one of the following criteria [recommended by the ICMJE (http://www.icmje.org/recomendations/)]:

- substantial contributions to conception and design, acquisition of data or analysis and interpretation of data;
- drafting the article or revising it critically for important intellectual content.

## Data Availability

Data sharing is not applicable to this article as no new data were created or analysed in this study.
